# Comparison of Anthropometric and Metabolic Indexes in the Diagnosis of Metabolic Syndrome: A Large-Scale Analysis of Spanish Workers

**DOI:** 10.3390/metabo15080495

**Published:** 2025-07-23

**Authors:** Juan José Guarro Miquel, Pedro Juan Tárraga López, María Dolores Marzoa Jansana, Ángel Arturo López-González, Pere Riutord Sbert, Carla Busquets-Cortés, José Ignacio Ramirez-Manent

**Affiliations:** 1 ADEMA-Health Group, University Institute for Research in Health Sciences (IUNICS), 07010 Palma, Spain; 23619jjg@comb.cat (J.J.G.M.); 24431dmj@comb.cat (M.D.M.J.); p.riutord@eua.edu.es (P.R.S.); c.busquets@eua.edu.es (C.B.-C.); joseignacio.ramirez@ibsalut.es (J.I.R.-M.); 2 Faculty of Medicine, UCLM (University of Castilla La Mancha), 02008 Albacete, Spain; pjtarraga@sescam.jccm.es; 3 SESCAM (Health Service of Castilla La Mancha), 02008 Albacete, Spain; 4 Faculty of Dentistry, ADEMA-Universidad de las Islas Baleares, 07010 Palma, Spain; 5 Balearic Islands Health Research Institute Foundation (IDISBA), 07010 Palma, Spain; 6 Balearic Islands Health Service, 07010 Palma, Spain; 7 Faculty of Medicine, University of the Balearic Islands, 07010 Palma, Spain

**Keywords:** metabolic syndrome, body mass index, TyG index, waist–triglyceride index

## Abstract

Background: Metabolic syndrome (MS) is a major public health concern linked to an elevated risk of type 2 diabetes and cardiovascular disease. Simple, reliable screening tools are needed for early identification, especially in working populations. Objective: To compare the diagnostic accuracy of body mass index (BMI), waist-to-height ratio (WtHR), triglyceride–glucose index (TyG), and waist–triglyceride index (WTI) for detecting MS based on NCEP ATP III and IDF criteria in a large cohort of Spanish workers. Methods: This cross-sectional study analyzed data from 386,924 Spanish workers. MS was diagnosed using NCEP ATP III and IDF definitions. The four indexes were evaluated by sex using a receiver operating characteristic (ROC) curve analysis. Area under the curve (AUC), optimal cut-off points, and Youden’s index were calculated. Results: TyG and WTI had the highest AUC values in men (0.911 and 0.901, respectively) for NCEP ATP III-defined MS, while WtHR and WTI achieved the best performance in women (0.955 and 0.953, respectively). WtHR outperformed BMI in all subgroups. Optimal cut-off values were identified according to sex and the definition of MS: TyG (8.95 men, 8.51 women), WtHR (0.54 men, 0.51 women), and WTI (170.6 men, 96.5 women), supporting their practical implementation in occupational health programs. All indexes showed significant discriminatory capacity (*p* < 0.001). Conclusions: TyG, WtHR, and WTI are more effective than BMI in detecting MS among Spanish workers, with sex-specific patterns. Their ease of use and diagnostic strength support their adoption in occupational health programs for early cardiometabolic risk detection.

## 1. Introduction

Metabolic syndrome (MS) is a cluster of interrelated cardiometabolic risk factors, including abdominal obesity, hypertension, dyslipidemia, and hyperglycemia, that significantly increase the risk of type 2 diabetes mellitus (T2DM), cardiovascular disease (CVD), and premature mortality [1]. As the prevalence of MS continues to rise globally, particularly in working-age populations, its early identification has become a critical objective in preventive healthcare strategies [2]. In Europe, estimates suggest that MS affects approximately 25% of adults, with significant geographic and sociodemographic variability [3].

The two most commonly used criteria for diagnosing MS are those proposed by the National Cholesterol Education Program Adult Treatment Panel III (NCEP ATP III) and the International Diabetes Federation (IDF). While both definitions include similar components, the IDF places greater emphasis on central obesity as a requisite criterion [4]. Consequently, the choice of definition may influence MS prevalence rates and its association with anthropometric and metabolic indicators.

Traditionally, body mass index (BMI) has been widely used as a surrogate marker for adiposity and cardiometabolic risk. However, BMI lacks the capacity to reflect fat distribution, particularly visceral adiposity, which plays a central role in the pathogenesis of MS [5]. Accordingly, waist-to-height ratio (WtHR) has emerged as a more reliable measure of central adiposity and metabolic risk across various populations [6].

Beyond anthropometric indicators, newer composite indexes such as the triglyceride–glucose (TyG) index and the waist–triglyceride index (WTI) have gained prominence due to their ability to capture both lipid and glycemic dysregulation, reflecting underlying insulin resistance [7,8]. The TyG index, in particular, has shown strong correlations with hyperinsulinemia and has been validated against the hyperinsulinemic-euglycemic clamp method [9]. Similarly, WTI integrates abdominal adiposity with triglyceride levels, offering a practical proxy for metabolic dysfunction [10].

Recent large-scale studies have assessed the diagnostic performance of these indexes in different populations. For instance, a cross-sectional analysis in Korean adults reported high sensitivity and specificity for the TyG index in detecting MS [11], while European cohorts have highlighted the superior predictive capacity of WtHR over BMI [12]. However, few studies have systematically compared the discriminatory capacity of these indicators within large, occupationally active European populations using both NCEP ATP III and IDF criteria.

Given the pressing need for simple, low-cost screening tools that can be implemented in workplace health programs, understanding which indexes best identify individuals at risk for MS is essential. This is particularly relevant for Spain, where occupational health surveillance covers a substantial proportion of the adult population, offering an opportunity for early detection and intervention [13].

The present study aimed to compare the diagnostic performance of four anthropometric and metabolic indexes—BMI, WtHR, TyG, and WTI—in identifying MS according to both NCEP ATP III and IDF criteria in a large cohort of Spanish workers. Additionally, we explored sex-specific differences and optimal cut-off points to inform clinical and public health strategies. By evaluating both traditional and novel markers within a real-world occupational context, this study contributes to the refinement of MS screening protocols and supports evidence-based policy development.

To our knowledge, this is the first study to simultaneously compare BMI, WtHR, TyG, and WTI for diagnosing MS in a large-scale European working population, with sex-stratified analysis and dual diagnostic criteria (NCEP ATP III and IDF). This unique approach enhances both the novelty and applicability of the findings. We selected these four indexes due to their widespread use, simplicity, and ability to reflect distinct dimensions of MS—adiposity (BMI, WtHR), and insulin resistance/lipid dysfunction (TyG, WTI).

## 2. Methods

### 2.1. Study Design and Population

This cross-sectional study analyzed data from a large cohort of Spanish workers who underwent routine occupational health assessments between 2021 and 2023. Participants were recruited from multiple companies across various economic sectors in Spain, including industry, services, and public administration. All participants provided informed consent prior to data collection, and the study protocol complied with the principles of the Declaration of Helsinki. The dataset was anonymized and approved by an independent ethics committee.

Inclusion criteria were: (i) age between 18 and 69 years, (ii) complete anthropometric, biochemical, and sociodemographic data, and (iii) no previously diagnosed cardiovascular or metabolic disease. Individuals with missing values for key variables such as triglycerides, fasting glucose, waist circumference, or height were excluded from the analysis.

### 2.2. Anthropometric and Biochemical Measurements

Trained personnel conducted all measurements following standardized protocols. Body weight and height were measured with calibrated electronic scales and stadiometers, respectively, and used to calculate body mass index (BMI) as weight in kilograms divided by height in meters squared (kg/m^2^). Waist circumference was measured at the midpoint between the lower rib and iliac crest. The waist-to-height ratio (WtHR) was calculated as waist circumference (cm) divided by height (cm). Blood pressure was recorded using an automated sphygmomanometer after a 5-min rest.

Fasting blood samples were drawn in the morning after an overnight fast of at least 8 h. Serum glucose, total cholesterol, HDL-cholesterol, LDL-cholesterol, and triglycerides were analyzed in accredited clinical laboratories using enzymatic methods.

The triglyceride–glucose (TyG) index was calculated as follows:

TyG = ln(triglycerides [mg/dL] × glucose [mg/dL]/2) [14]

The waist–triglyceride index (WTI) was defined as

WTI = waist circumference [cm] × triglycerides [mg/dL] [15]

### 2.3. Definition of Metabolic Syndrome

Metabolic syndrome (MS) was diagnosed using two internationally recognized criteria: the National Cholesterol Education Program Adult Treatment Panel III (NCEP ATP III) [16] and the International Diabetes Federation (IDF) [17].

According to the NCEP ATP III, MS was defined by the presence of three or more of the following criteria:

- Waist circumference ≥102 cm (men) or ≥88 cm (women)
- Triglycerides ≥150 mg/dL
- HDL-cholesterol <40 mg/dL (men) or <50 mg/dL (women)
- Blood pressure ≥130/85 mmHg or on antihypertensive treatment
- Fasting glucose ≥100 mg/dL or diagnosed diabetes

The IDF criteria require the presence of central obesity (waist circumference ≥94 cm in men or ≥80 cm in women, for European populations) plus any two of the remaining four components.

### 2.4. Lifestyle and Sociodemographic Variables

Information on physical activity, smoking, Mediterranean diet adherence, age, sex, and occupational social class was obtained through structured questionnaires. Physical activity was determined using the standardized IPAQ questionnaire [18]. Adherence to the Mediterranean diet was assessed using a brief dietary screener validated in the Spanish population [19]. Social class was classified into I (high), II (intermediate), and III (manual workers) according to the Spanish adaptation of the British Registrar General’s classification [20].

### 2.5. Statistical Analysis

Continuous variables were presented as mean ± standard deviation (SD) and categorical variables as absolute and relative frequencies. Between-group comparisons were performed using the Student’s *t*-test or Mann–Whitney U test for continuous variables and χ^2^ test for categorical variables.

To assess the discriminatory ability of BMI, WtHR, TyG, and WTI for MS, a receiver operating characteristic (ROC) curve analysis was conducted. Area under the curve (AUC) values were calculated with 95% confidence intervals (CI), and the optimal cut-off values were identified using Youden’s index. Analyses were stratified by sex and MS definition (NCEP ATP III and IDF). AUC values were interpreted as follows: 0.5–0.6 = poor; 0.6–0.7 = fair; 0.7–0.8 = acceptable; 0.8–0.9 = excellent; >0.9 = outstanding [21].

All statistical analyses were performed using SPSS version 29.0 (IBM Corp., Armonk, NY, USA). A two-tailed *p*-value < 0.05 was considered statistically significant.

**Figure 1:**
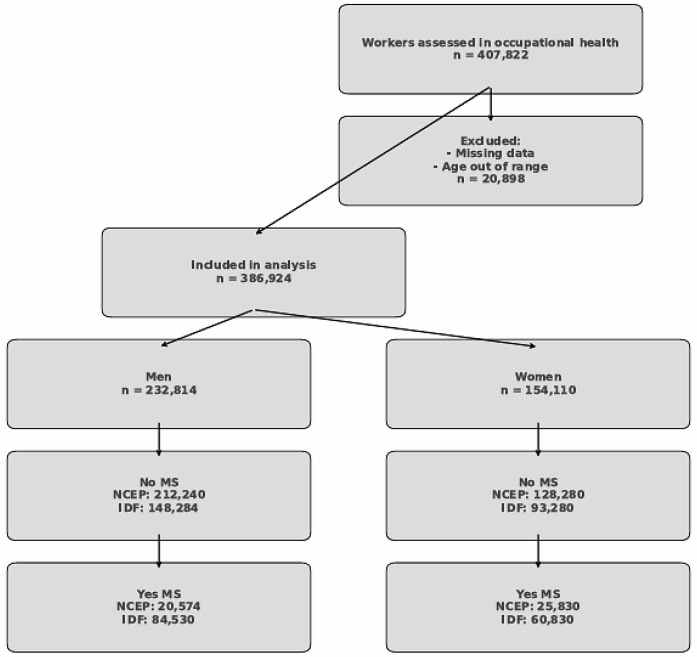
Flowchart of study population selection and classification by sex and metabolic syndrome status.

This flowchart outlines the selection and classification process of the study population. A total of 407,822 Spanish workers undergoing occupational health assessments were initially considered for analysis. After excluding 20,898 individuals due to missing data or age criteria outside the predefined range, 386,924 participants were retained for the final analysis (Figure 1).

The analytical cohort was subsequently stratified by sex: 232,814 men and 154,110 women. Each sex-specific subgroup was further divided based on the presence or absence of metabolic syndrome (MS) according to two established diagnostic criteria: the National Cholesterol Education Program Adult Treatment Panel III (NCEP ATP III) and the International Diabetes Federation (IDF).

Among men, 212,240 were classified as free of MS according to the NCEP criteria, whereas 20,574 met the diagnostic threshold for MS. In contrast, using the IDF criteria, only 148,284 men were considered metabolically healthy, while a larger subset (84,530 men) fulfilled the MS definition, reflecting the greater sensitivity of the IDF criteria.

Among women, 128,280 did not meet the NCEP criteria for MS, while 25,830 did. When applying the IDF definition, 93,280 women were considered free of MS, whereas 60,830 were classified as having MS. These differences underscore the variability in MS prevalence depending on the diagnostic definition applied, particularly among women.

This structured selection and classification allowed for a robust comparative analysis of anthropometric and metabolic indexes in relation to metabolic syndrome diagnosis, with stratification by sex and MS definition enhancing the interpretability and clinical relevance of the findings.

## 3. Results

Table 1 presents a comprehensive overview of the baseline sociodemographic, anthropometric, clinical, and lifestyle characteristics of the study population, stratified by sex. Statistically significant differences were observed between men (*n* = 232,814) and women (*n* = 154,110) across nearly all variables (*p* < 0.001), underscoring notable sex-specific patterns relevant to cardiometabolic risk.

Men exhibited significantly higher values in height, weight, waist circumference, and systolic and diastolic blood pressure. Additionally, they had higher mean levels of triglycerides and fasting glucose, whereas women showed higher HDL-cholesterol and slightly higher LDL-cholesterol values. These differences align with known physiological variations and their implications for differential metabolic syndrome (MS) risk profiles.

Regarding age distribution, the cohorts were similarly composed, with the highest representation in the 30–39 and 40–49-year age groups. Educational attainment and occupational social class also varied significantly: a greater proportion of men had had only a primary education and belonged to social class III (manual workers), whereas women were more likely to have had a university education and belong to higher social classes (I and II). These findings reflect underlying socioeconomic disparities that may influence health outcomes.

Lifestyle habits further differentiated the sexes. Women reported higher levels of physical activity and greater adherence to the Mediterranean diet, both protective factors against MS. Moreover, smoking prevalence was significantly higher among men. Such behavioral differences are critical to consider when interpreting the prevalence and risk stratification of metabolic disorders.

Overall, Table 1 highlights clear sex-based differences in metabolic and lifestyle determinants. These findings support the rationale for stratified analyses in the diagnostic assessment of metabolic syndrome and suggest the need for tailored public health strategies that address both biological and sociocultural determinants of cardiometabolic health.

Table 2 evaluates four key anthropometric and metabolic indexes—BMI, waist-to-height ratio (WtHR), triglyceride–glucose index (TyG), and waist–triglyceride index (WTI)—across MS status and sex. Across all indexes, subjects with MS exhibited significantly higher mean values compared to those without MS (*p* < 0.001). Among men and women alike, BMI and WtHR were considerably elevated in MS-positive groups, especially under IDF criteria, which tend to identify more cases due to lower thresholds.

Importantly, the proportion of individuals exceeding standard cut-off values was also notably higher in the MS groups. For example, over 90% of women with MS (IDF) had a high WtHR, and more than 80% of men with MS (NCEP ATP III) had a high TyG index. These findings suggest a strong discriminatory potential of these indexes in identifying MS, particularly the TyG and WTI, which reflect combined dyslipidemic and glycemic burden.

**Figure 2:**
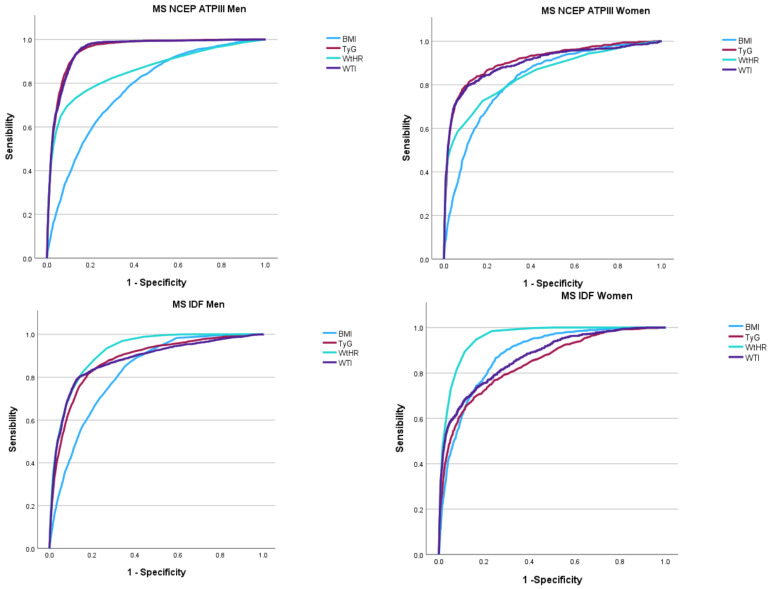
Area under the curve (AUC) of BMI, WtHR, TyG, and WTI for identifying metabolic syndrome based on NCEP ATP III and IDF criteria in men and women. MS: Metabolic syndrome. NCEP ATPII: National Cholesterol Education Program Adult Treatment Panel III. IDF: International Diabetes Federation.

Figure 2 graphically summarizes the area under the curve (AUC) of the four indexes (BMI, WtHR, TyG, and WTI) for identifying MS by sex and definition. The visual comparison underscores the superior diagnostic performance of TyG and WTI in men and of WtHR and WTI in women, especially under the NCEP ATP III criteria. The graphical trends support the tabular findings and emphasize the potential of using simple, calculated indexes for an early detection of MS in large populations.

Table 3 displays the diagnostic performance of BMI, WtHR, TyG, and WTI in identifying MS according to receiver operating characteristic (ROC) curve analyses. For men, the TyG index showed the highest AUC (0.911) for MS (NCEP ATP III), followed closely by WTI (0.901), indicating an excellent discriminatory capacity. In women, the highest AUCs were for WtHR (0.955) and WTI (0.953) under the NCEP ATP III definition, suggesting a superior performance of central adiposity-based indexes.

For MS defined by IDF, WtHR had the best performance in both sexes (AUC = 0.919 in men and 0.955 in women), reinforcing its utility as a screening tool. The optimal cut-off values, with balanced sensitivity and specificity, were also provided for each index, offering practical guidance for clinical use.

Optimal cut-off values were identified as follows: TyG (8.95 men, 8.51 women), WtHR (0.54 men, 0.51 women), and WTI (170.6 men, 96.5 women), supporting their practical implementation in occupational health programs.

As the AUCs were very similar, we used DeLong’s test to evaluate statistical differences (Supplementary Material). Pairwise comparisons using DeLong’s test revealed statistically significant differences between most AUCs (*p* < 0.001), confirming the superior diagnostic accuracy of TyG and WTI in men, and WtHR and WTI in women, particularly under the NCEP ATP III criteria.

This statistical confirmation reinforces our findings that TyG and WTI—indexes integrating metabolic and central adiposity components—outperform traditional measures like BMI in men. Similarly, the superior performance of WtHR and WTI in women suggests that central fat distribution, more than general adiposity or lipid–glucose parameters alone, may be key in identifying metabolic syndrome risk in this group.

## 4. Discussion

This large-scale cross-sectional study evaluated and compared the diagnostic capacity of four anthropometric and metabolic indexes—BMI, WtHR, TyG, and WTI—for identifying metabolic syndrome (MS) based on NCEP ATP III and IDF definitions in a large occupational cohort of Spanish workers. We selected these four indexes due to their widespread use, simplicity, and ability to reflect distinct dimensions of MS—adiposity (BMI, WtHR), and insulin resistance/lipid dysfunction (TyG, WTI). While derivatives such as TyG-BMI or TyG-WHtR have shown promise [22], our goal was to focus on core markers to maximize translational value. Future work will expand to include these advanced indexes. The results demonstrate that indexes reflecting central adiposity and insulin resistance (particularly WtHR, TyG, and WTI) outperform BMI in distinguishing individuals with MS, with slight variations by sex and diagnostic criteria.

Among men, TyG and WTI were the most effective tools, with AUCs exceeding 0.90, while WtHR and WTI showed superior performance in women, especially under IDF criteria. These findings are consistent with recent evidence highlighting the TyG index as a powerful evaluator of adverse cardiometabolic outcomes, such as metabolic syndrome, coronary heart disease, and type 2 diabetes [23,24].

The outstanding AUCs (>0.9) observed may partially reflect the large sample size, which increases statistical power and reduces variability. Such performance may not be replicated in smaller cohorts.

The WTI, a novel index combining waist circumference and triglyceride levels, also showed high diagnostic accuracy across sex and diagnostic criteria. Studies have confirmed its usefulness in capturing the combined effects of abdominal adiposity and dyslipidemia, particularly in Mediterranean and East Asian populations [24,25]. In the present study, WTI achieved comparable or superior AUC values to TyG, emphasizing its potential as a robust and practical marker for workplace screening.

WtHR also proved highly effective, particularly in women, where it exceeded an AUC of 0.95 under IDF criteria. This aligns with recent findings from the SIMETAP-AO study, which demonstrated the strong association between high WtHR and cardiometabolic abnormalities in Spanish primary care settings [25]. Importantly, WtHR is simple to obtain, requires only two anthropometric measurements, and does not depend on blood testing—making it especially advantageous for large-scale screening.

By contrast, although BMI remains widely used, its performance was consistently lower than that of the other indexes. BMI fails to capture fat distribution or distinguish between lean and fat mass, which limits its sensitivity in identifying individuals at cardiometabolic risk. Our findings are in line with several recent investigations reporting that BMI-based classifications may underestimate risk in individuals with central obesity or metabolic dysfunction [26].

Sex differences were also evident. Men generally exhibited higher TyG and WTI values and had stronger associations between these indexes and MS. Conversely, WtHR and BMI performed better in women. These differences likely reflect distinct patterns in fat distribution, insulin sensitivity, and hormonal regulation. Women are more likely to accumulate subcutaneous fat, which is metabolically less harmful, while men tend to exhibit greater visceral adiposity, closely linked to insulin resistance [27].

Although stratified analyses were primarily based on significant sex-based physiological and metabolic differences, future studies should consider global models with interaction terms and subsequent stratification by both sex and age to improve applicability and parsimony in clinical settings.

The TyG index, calculated from fasting triglycerides and glucose, is a recognized surrogate of insulin resistance. Its strong correlation with clamp-derived insulin sensitivity has been repeatedly demonstrated [23,26], and it has recently been associated with incident cardiovascular events in hypertensive and coronary populations [28]. In our study, TyG consistently outperformed BMI and showed comparable results to WTI, reinforcing its clinical value as a screening tool.

Similarly, recent studies have identified WTI as a promising index for assessing the risk of developing metabolic syndrome and chronic kidney disease [29,30]. Its simplicity, combining routine measurements (waist circumference and triglycerides), makes it highly applicable in occupational health programs and routine primary care settings. Notably, WTI avoids the need for insulin measurements, often unavailable in large-scale or resource-limited screenings.

Differences in metabolic levels between physical and non-physical laborers within sex groups may contribute to variability in MS risk [31]. Although occupational type was recorded, detailed stratified analyses by job activity were beyond this study’s scope. Future research should evaluate how occupational physical demands modulate the predictive capacity of metabolic indices.

From a public health perspective, the results of this study provide compelling evidence for the incorporation of WtHR, TyG, and WTI into workplace health assessments, especially considering their simplicity, reproducibility, and superior predictive value compared to BMI. Previous studies have emphasized the benefit of using non-invasive or low-cost metrics to detect high-risk individuals early and initiate preventive strategies [32,33].

The proposed cut-off values are easily derived from routine health checks, making them practical for workplace-based screenings. Their implementation could enhance early detection and personalized interventions for MS risk, especially in resource-limited settings.

## 5. Strengths

The strengths of the study include the use of a very large, representative cohort that was thoroughly characterized both clinically and sociodemographically.

Another notable strength of the study is the sex-stratified analysis, along with the application of two validated and widely recognized definitions of metabolic disease.

In addition, the inclusion of multiple validated indices in a single comparative analysis not only enriches the interpretation of the findings but also considerably enhances the practical applicability of our results.

## 6. Limitations

One of the main limitations of this study is its cross-sectional design, which precludes any causal inference. Since data were collected at a single point in time, it is not possible to determine the temporal sequence between exposure and outcome variables, nor to exclude the potential influence of unmeasured confounding factors.

Given the occupational nature of the cohort, a healthy worker effect cannot be ruled out, which may lead to an underestimation of MS prevalence and limit generalizability to high-risk or non-working populations. This may also influence the optimal cut-off values reported and potentially overestimate the diagnostic performance of the evaluated indices.

While we identified optimal cut-off points for each index, these require validation in external and longitudinal cohorts.

Although direct insulin resistance measures (e.g., HOMA-IR or euglycemic-hyperinsulinemic clamp) were unavailable, the TyG index has demonstrated strong correlation with these gold standards, making it a validated surrogate marker [22,23]. However, we acknowledge that the absence of these reference methods may limit a direct comparison of metabolic indexes.

We did not perform age-specific stratification within sex groups, which could have revealed age-related variability in index performance. Future analyses should evaluate whether diagnostic cut-offs or index effectiveness vary across age strata.

Although cost-effectiveness was not directly assessed, all four indexes require minimal resources and are highly feasible in occupational settings. Formal economic evaluations should be undertaken in future research.

## 7. Conclusions

In conclusion, this study confirms that indexes reflecting central adiposity and metabolic dysregulation—particularly TyG, WtHR, and WTI—outperform BMI in identifying individuals with metabolic syndrome in a large sample of Spanish workers. TyG and WTI were the most accurate in men, while WtHR and WTI performed best in women.

Sex differences may reflect hormonal modulation of fat deposition and insulin sensitivity. Women generally exhibit more subcutaneous fat and estrogen-mediated insulin sensitivity, favoring the performance of central adiposity-based indexes like WtHR. Conversely, men accumulate more visceral fat, amplifying the utility of lipid–glucose indexes such as TyG and WTI.

These indexes serve as effective tools for identifying prevalent cases of MS in occupational settings. Given the cross-sectional design, their predictive value for future cardiometabolic outcomes requires further longitudinal validation.

## Figures and Tables

**Table 1:** Baseline sociodemographic, clinical, and lifestyle characteristics of Spanish workers by sex.

	Men *n* = 232,814	Women *n* = 154,110	
	Mean (SD)	Mean (SD)	*p*-Value
Age (years)	39.8 (10.3)	39.2 (10.2)	<0.001
Height (cm)	173.9 (7.0)	161.2 (6.6)	<0.001
Weight (kg)	81.1 (13.9)	65.3 (13.2)	<0.001
Waist circumference (cm)	87.7 (9.1)	73.9 (7.9)	<0.001
Hip circumference (cm)	100.0 (8.4)	97.2 (8.9)	<0.001
Systolic blood pressure (mmHg)	124.4 (15.1)	114.4 (14.8)	<0.001
Diastolic blood pressure (mmHg)	75.4 (10.6)	69.7 (10.3)	<0.001
Total cholesterol (mg/dL)	195.9 (38.9)	193.6 (36.4)	<0.001
HDL-c (mg/dL)	51.0 (7.0)	53.7 (7.6)	<0.001
LDL-c (mg/dL)	120.5 (37.6)	122.3 (37.0)	<0.001
Triglycerides (mg/dL)	123.8 (88.0)	88.1 (46.2)	<0.001
Glycaemia (mg/dL)	88.1 (12.9)	84.1 (11.5)	<0.001
	%	%	*p*-value
20–29 years	17.9	19.5	<0.001
30–39 years	33.1	33.3	
40–49 years	29.7	29.4	
50–59 years	16.3	15.3	
60–69 years	3.0	2.5	
Primary school	61.2	51.8	<0.001
Secondary school	34.0	40.7	
University	4.8	7.5	
Social class I	5.3	7.2	<0.001
Social class II	17.4	33.2	
Social class III	77.3	59.8	
Non-physical activity	54.5	47.8	<0.001
Yes physical activity	45.5	52.2	
Non-Mediterranean diet	59.0	48.6	<0.001
Yes Mediterranean diet	41.0	51.4	
Non-smokers	62.9	67.0	<0.001
Smokers	37.1	33.0	

HDL: High density lipoprotein. LDL: Low density lipoprotein. SD: Standard deviation.

**Table 2:** Distribution of anthropometric and metabolic indexes and the prevalence of abnormal values by sex and metabolic syndrome status.

	No MS NCEP ATPIII	Yes MS NCEP ATPIII		No MS IDF	Yes MS IDF	
	*n* = 212,240	*n* = 20,574		*n* = 148,284	*n* = 5826	
Men	Mean (SD)	Mean (SD)	*p*-value	Mean (SD)	Mean (SD)	*p*-value
BMI	26.4 (3.9)	30.7 (4.7)	<0.001	26.2 (3–8)	31.2 (4.3)	<0.001
WtHR	0.50 (0.05)	0.59 (0.06)	<0.001	0.50 (0.04)	0.59 (0.05)	<0.001
TyG	8.3 (0.5)	9.4 (0.4)	<0.001	8.3 (0.5)	9.2 (0.6)	<0.001
WTI	107.1 (65.8)	325.4 (183.4)	<0.001	106.6 (66.7)	277.4 (182.1)	<0.001
Women	Mean (SD)	Mean (SD)	*p*-value	Mean (SD)	Mean (SD)	*p*-value
BMI	24.9 (4.7)	31.8 (6.2)	<0.001	24.8 (4.6)	33.2 (5.8)	<0.001
WtHR	0.45 (0.05)	0.54 (0.07)	<0.001	0.45 (0.04)	0.56 (0.05)	<0.001
TyG	8.1 (0.4)	9.0 (0.5)	<0.001	8.1 (0.5)	8.8 (0.5)	<0.001
WTI	70.5 (35.3)	184.0 (101.2)	<0.001	70.5 (35.5)	170.5 (102.5)	<0.001
	No MS NCEP ATPIII	Yes MS NCEP ATPIII		No MS IDF	Yes MS IDF	
Men	%	%	*p*-value	%	%	*p*-value
BMI obesity	16.3	52.7	<0.001	13.5	58.5	<0.001
WtHR high	43.9	87.5	<0.001	14.1	68.1	<0.001
TyG high	17.6	96.0	<0.001	8.4	77.9	<0.001
Women	%	%	*p*-value	%	%	*p*-value
BMI obesity	14.8	55.9	<0.001	12.9	67.9	<0.001
WtHR high	41.1	98.6	<0.001	12.8	91.6	<0.001
TyG high	17.2	80.1	<0.001	8.8	60.5	<0.001

MS: Metabolic syndrome. NCEP ATPII: National Cholesterol Education Program Adult Treatment Panel III. IDF: International Diabetes Federation. BMI: Body mass index. WtHR: Waist-to-height ratio. TyG: Triglyceride–glucosa index. WTI: Waist–Triglyceride index.

**Table 3:** Diagnostic accuracy of anthropometric and metabolic indexes for metabolic syndrome (NCEP ATP III and IDF) according to sex: ROC curve analysis.

	Men		Women	
MS NCEP ATPIII	AUC (95% CI)	Cut-Off-Sensibility-Specificity-Youden	AUC (95% CI)	Cut-Off-Sensibility-Specificity-Youden
BMI	0.825 (0.820–0.830)	28.0-70.7-70.2-0.409	0.775 (0.772–0.778)	27.3-75.8-74.7-0.505
WtHR	0.846 (0.840–0.852)	0.54-80.9-73.5-0.544	0.858 (0.855–0.861)	0.49-76.2-75.5-0.517
TyG	0.911 (0.907–0.916)	8.95-90.1-89.0-0.791	0.954 (0.953–0.955)	8.51-83.9-83.9-0.678
WTI	0.901 (0.895–0.906)	170.6-89.0-89.0-0.780	0.953 (0.952–0.955)	96.5-83.4-83.0-0.664
MS IDF	AUC (95% CI)	Cut-off-sensibility-specificity-Youden	AUC (95% CI)	Cut-off-sensibility-specificity-Youden
BMI	0.822 (0.820–0.824)	28.2-73.9-73.5-0.474	0.880 (0.876–0.883)	28.0-79.5-78.9-0.584
WtHR	0.919 (0.918–0.921)	0.54-85.0-82.2-0.672	0.955 (0.953–0.957)	0.51-89.3-88.7-0.780
TyG	0.880 (0.877–0.882)	8.78-81.9-81.6-0.635	0.844 (0.838–0.849)	8.38-76.8-75.6-0.524
WTI	0.879 (0.877–0.882)	144.2-82.1-82.0-0.641	0.871 (0.866–0.875)	88.3-77.3-77.3-0.546

MS: Metabolic syndrome. NCEP ATPII: National Cholesterol Education Program Adult Treatment Panel III. IDF: International Diabetes Federation. BMI: Body mass index. WtHR: Waist-to-height ratio. TyG: Triglyceride–glucosa index. WTI: Waist–Triglyceride index. AUC: Area under the curve.

## Data Availability

The data collected and analyzed in this study are securely stored within a restricted-access database managed by ADEMA University School. All data handling complies with institutional and legal data protection requirements, under the supervision of the designated Data Protection Officer, Dr. Ángel Arturo López González.

## Supplementary Materials

The following supporting information can be downloaded at: https://www.mdpi.com/article/10.3390/metabo15080495/s1, Table S1. Pairwise comparison of areas under the curve (AUC) for the diagnosis of metabolic syndrome using the DeLong test, stratified by sex and diagnostic criteria (NCEP ATP III and IDF).
