# Supplementary material for: Comparison of Anthropometric and Metabolic Indexes in the Diagnosis of Metabolic Syndrome: A Large-Scale Analysis of Spanish Workers
Source: Metabolites. 2025 Jul 23;15(8):495. doi: 10.3390/metabo15080495 (PMC12388431; doi:10.3390/metabo15080495)
Supplement: Supplementary file 1 [file metabolites-15-00495-s001.zip › metabolites-3717543-Supplementary.pdf]

Supplementary Materials:

Supplementary Table S1. Pairwise comparison of areas under the curve (AUC) for the diagnosis of metabolic syndrome using the DeLong test, stratified by sex and diagnostic criteria (NCEP ATP III and IDF).

| Sex | SM Criterion | Comparative indices | AUC 1 | AUC 2 | p-value (DeLong) |
|---|---|---|---|---|---|
| Men | NCEP ATP III | TyG vs. WTI | 0.911 | 0.901 | <0.001 |
| Men | NCEP ATP III | TyG vs. WtHR | 0.911 | 0.846 | <0.001 |
| Men | NCEP ATP III | TyG vs. IMC | 0.911 | 0.825 | <0.001 |
| Men | NCEP ATP III | WTI vs. WtHR | 0.901 | 0.846 | <0.001 |
| Men | NCEP ATP III | WTI vs. IMC | 0.901 | 0.825 | <0.001 |
| Men | NCEP ATP III | WtHR vs. IMC | 0.846 | 0.825 | <0.001 |
| Women | NCEP ATP III | WtHR vs. WTI | 0.955 | 0.953 | 0.012 |
| Women | NCEP ATP III | WtHR vs. TyG | 0.955 | 0.954 | 0.086 |
| Women | NCEP ATP III | WtHR vs. IMC | 0.955 | 0.775 | <0.001 |
| Women | NCEP ATP III | WTI vs. TyG | 0.953 | 0.954 | 0.221 |
| Women | NCEP ATP III | WTI vs. IMC | 0.953 | 0.775 | <0.001 |
| Women | NCEP ATP III | TyG vs. IMC | 0.954 | 0.775 | <0.001 |
| Men | IDF | WtHR vs. TyG | 0.919 | 0.880 | <0.001 |
| Men | IDF | WtHR vs. WTI | 0.919 | 0.879 | <0.001 |
| Men | IDF | WtHR vs. IMC | 0.919 | 0.822 | <0.001 |
| Women | IDF | WtHR vs. TyG | 0.955 | 0.844 | <0.001 |
| Women | IDF | WtHR vs. WTI | 0.955 | 0.871 | <0.001 |
| Women | IDF | WtHR vs. IMC | 0.955 | 0.880 | <0.001 |

Note: AUC: Area under the curve. BMI: Body mass index. WtHR: Waist-to-height ratio. TyG: Triglyceride-glucose ratio. WTI: Waist-to-triglyceride ratio. Comparisons were made using De-Long's test for correlated ROC curves. P values < 0.05 indicate statistically significant differences.
